# Parasite cloud service providers: on-demand prices on top of spot prices

**DOI:** 10.1016/j.heliyon.2019.e02877

**Published:** 2019-11-28

**Authors:** Hamid Haghshenas, Jafar Habibi, Mohammad Amin Fazli

**Affiliations:** Computer Engineering Department, Sharif University of Technology, Azadi Avenue, Tehran, Iran

**Keywords:** Computer science, Cloud computing, Spot price, Martingale, Service Level Agreement, Dynamic load

## Abstract

On-demand resource provisioning and elasticity are two of the main characteristics of the cloud computing paradigm. As a result, the load on a cloud service provider (CSP) is not fixed and almost always a number of its physical resources are not used, called spare resources. As the CSPs typically don't want to be overprovisioned at any time, they procure physical resources in accordance to a pessimistic forecast of their loads and this leads to a large amount of spare resources most of the time. Some CSPs rent their spare resources with a lower price called the spot price, which varies over time with respect to the market or the internal state of the CSP. In this paper, we assume the spot price to be a function of the CSP's load. We introduce the concept of a parasite CSP, which rents spare resources from several CSPs simultaneously with spot prices and rents them to its customers with an on-demand price lower than the host CSPs' on-demand prices. We propose the overall architecture and interaction model of the parasite CSP. Mathematical analysis has been made to calculate the amount of spare resources of the host CSPs, the amount of resources that the parasite CSP can rent (its virtual capacity) as well as the probability of SLA violations. We evaluate our analysis over pricing data gathered from Amazon EC2 services. The results show that if the parasite CSP relies on several host CSPs, its virtual capacity can be considerable and the expected penalty due to SLA violation is acceptably low.

## 1 Introduction

Rapid growth of the Internet has encouraged the evolution of new technological concepts and paradigms, cloud computing being among them. Using this paradigm, enterprises can have access to infrastructures without much hassle and spending lots of effort and cost on the management issues. Moreover, computing resources which have become cheaper and more powerful, can be employed without the need to plan ahead for provisioning. Cloud computing gains benefits from different concepts such as distributed computing, grid computing, and parallel computing [1] and has realized many computer scientists' dream of using computing resources as a utility. As a result, these resources can be leased and released in an on-demand fashion and with a usage-based pricing model through the Internet [2].

Cloud service providers (CSPs) usually argue their capacity to be unlimited and it's not their desire to reject a customer's request due to lack of resources. Hence, the CSPs typically procure their physical resources in order to handle the demand in peak conditions. As a result, a considerable amount of physical resources are unused most of the time, which are called *spare resources*. An idea leveraged by a number of today service providers is to rent the spare resources with a price lower than the on-demand price. This price is called the *spot price* and varies over time regarding to the market and the internal state of the CSP. Here, we assume the CSP's spot price to be a function of its load. A user presents a bid to the CSP and can use the spare resources while the spot price is less than the bid.

Although the spot price is usually less than the on-demand price, unavailability of resources due to the spot price exceeding the bid is a risk. In addition, variability of the spot price makes the long-term costs unpredictable for the users. Hence, many users prefer to use the always-available reserved resources with the fixed on-demand price. The goal of this paper is to let the users use spare resources with high availability and at a fixed price in order to decrease the mentioned risks for them.

Imagine a user is going to use cloud resources provided by a cloud service provider. After investigating different price schemes, the user finds himself in a trade-off between price, availability and predictability. If he chooses to use spot prices to reduce the costs, a risk of being unavailable arises and also the future costs will not be predictable. What if the user can use the spot prices in a highly available manner and with a fixed price?

In this paper, we introduce the concept of a *parasite CSP*, which is in fact a virtual CSP exposing resources to its customers with an on-demand price lower than that of the normal CSP (called the *host CSP*). A parasite CSP has no physical resources by its own, but rents a number of resources from some host CSPs with their varying spot prices and rents them to its customers with a fixed on-demand price.

The main contributions of this paper include:

1. Introducing the concept of a parasite CSP which is a new concept to our best of knowledge
2. Proposing the overall architecture and interaction model of a parasite CSP
3. Mathematical analysis on the amount of spare resources in host CSPs and the virtual capacity of the parasite CSP which relies on several host CSPs
4. Mathematical analysis on the SLA of the parasite CSP, the probability of SLA violation and upper bounds for the expected penalty

In section 2 we express the problem context, introduce our basic idea and propose an overall architecture as well as interaction model. Mathematical analysis on the host CSPs' loads, the parasite CSP's virtual capacity and its SLA is formulated in section 3 and evaluated in section 4. Section 5 summarizes the previous related research and finally, section 6 concludes the paper.

## 2 The proposed model

### 2.1 Pricing model

A CSP (which we call it the *host CSP*) owns a large number of physical resources and a virtualization software installed on them. Customers request virtual machines (VMs) in an on-demand manner and the CSP creates and rents VMs to them. Let ${\mathrm{pr}}_{o}$ be the on-demand price per resource per time unit.

However, the demand of the CSP is not constant and varies over time. On the one hand, the CSP exhibits an infinite capacity to its customers and it should not reject the requests due to a lack in its physical resources. So the amount of available physical resources should not be less than the total demand most of the time. On the other hand, a considerable amount of physical resources may be free in each time, charging the CSP for maintenance costs but gaining no profit because they are not used for hosting customer VMs. These resources are called *spare resources*.

The CSP can rent the spare resources by a price different from the on-demand price, called the *spot price*. We assume this price to depend on the current load of the CSP; the lower the load of the CSP, the lower the spot price of the spare resources. Since dealing with real numbers (not necessarily integers) are simpler in this context, we assume the current load of the CSP and the spot price to be real numbers.

Definition 1 **Spot price**

A strictly increasing function $\mathrm{spot}:R^{\geq 0}\to R^{+}$ where spot(l) denotes the spot price per resource if the current load of the CSP is *l*. This function is invertible and its inverse is denoted by ${\mathrm{spot}}^{-1}:R^{+}\to R^{\geq 0}$.

Let load(t) denote the total load of the CSP at time $t\in Z^{\geq 0}$, which equals the load for the on-demand requests (${\mathrm{load}}_{o}(t)$) plus the load for the requested spare resources (${\mathrm{load}}_{s}(t)$):

$$\mathrm{load}(t)={\mathrm{load}}_{o}(t)+{\mathrm{load}}_{s}(t)$$

The spot price per resource in time interval $\left[t_{1},t_{2}\right)$ equals

$$\sum_{t=t_{1}}^{t_{2}-1}\mathrm{spot}\;\left({\mathrm{load}}_{o}(t)+{\mathrm{load}}_{s}(t)\right)$$

and the CSP's revenue equals

$$\sum_{t=t_{1}}^{t_{2}-1}\left[{\mathrm{pr}}_{o}\;{\mathrm{load}}_{o}(t)+\mathrm{spot}\;\left({\mathrm{load}}_{o}(t)+{\mathrm{load}}_{s}(t)\right){\mathrm{load}}_{s}(t)\right]$$

Note that the CSP may violate its SLA occasionally and the paid penalty should be subtracted from the above amount. However, we ignore it now for simplicity and consider it later in this paper.

In order to use the spare resources, a customer sends a request to the CSP consisting a demand *v* and a bid *π* which is a price threshold. The CSP allocates an amount of *v* spare resources (if available) and keeps these resources available to the customer while the spot price is no more than *π*. If load(t) is the load of the CSP except the load added by this customer, then the price that the customer pays to the CSP in time interval $\left[t_{1},t_{2}\right)$ is

$$\sum_{t=t_{1}}^{t_{2}-1}\mathrm{spot}(\mathrm{load}(t)+v)v$$

### 2.2 The basic idea

In the last subsection, we introduced the concept of spot prices via which a CSP can rent its spare resources. In this subsection, we will see how an external player can leverage the spot prices to gain profit.

A *parasite CSP* is a CSP that owns no physical resource by itself. It exposes computational resources to its customers by an on-demand price of ${\mathrm{pr}}_{p}$ and uses the spare resources of one or several actual CSPs (the host CSPs) in the back. If an amount of *v* resources are used by the parasite CSP in time interval $\left[t_{1},t_{2}\right)$ and only one host CSP exists, the profit of the parasite CSP will be

$$(t_{2}-t_{1}){\mathrm{pr}}_{p}\;v-\sum_{t=t_{1}}^{t_{2}-1}\mathrm{spot}(\mathrm{load}(t)+v)v$$

If the spot price of the host CSP exceeds the bid *π*, the parasite CSP becomes unavailable and may violate its SLA. In fact, the unavailability of the parasite CSP may be due to the host CSP being unavailable or its spot price being above *π*. If both the host CSP and parasite CSP violate their SLAs, the host CSP pays some penalty to the parasite CSP and it pays some penalty to the customer in turn. If the host CSP does not violate its CSP but the parasite CSP, only the latter will pay some penalty to the customer. We have ignored these penalties in this subsection's formulations and will take them into account later in our mathematical analysis (subsection 3.4).

### 2.3 Architecture

**Figure 1:**
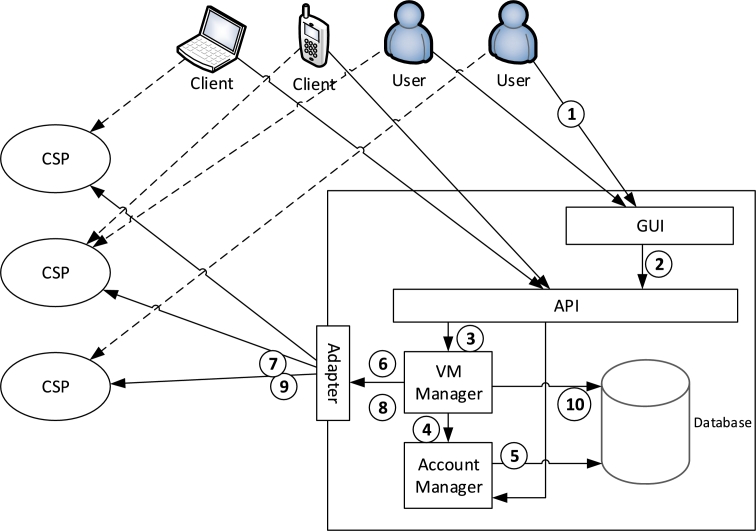
The internal architecture and interaction model of the parasite CSP.

Fig. 1 shows a high-level architectural model of the parasite CSP and its interactions with its customers and the host CSPs. In the figure, solid lines are interactions used for management commands and dashed lines are interactions used by the customers to access their VMs directly.

The main components of the parasite CSP are described below:

• **user interface (GUI):** a web-based component which the customers can interact to via their browsers
• **Application programming interface (API):** an interface for non-human clients on PCs, smart phones, etc. to interact to the parasite CSP. The GUI component also sends customer's requests to this component
• **VM manager:** responsible for managing the VMs of the customers. This component receives VM creation, resizing or deletion requests from the API and interacts to the host CSPs via appropriate adapters
• **Adapter:** responsible for communicating to different CSPs via their own APIs. In fact, exposes a unified interface for the parasite CSP and encapsulates the individual APIs of the host CSPs
• **Account manager:** a component that manages customers' budgets. Charging the customers' accounts and updating their budget during their use of VMs is the main responsibility of this component
• **Database:** persists customer-VM assignments as well as the customer's budgets and related information (VM prices, customer payments, etc.)

The numbers over the interactions demonstrate the order of interactions performed for creating a new VM (return messages are not shown in the figure):

1. The customer asks the parasite CSP via the GUI to create a new VM
2. The GUI sends an appropriate request to the API
3. The API asks VM manager to create a new VM for the customer
4. The VM manager asks the accounts manager component to ensure that the customer has enough budget in order to create a new VM
5. The account manager retrieves the customer's account information from the database and returns the result to VM manager
6. The VM manager asks the adapter component to retrieve current spot prices of different CSPs
7. The adapter sends individual requests to the host CSPs and asks their current spot price. The adapter then gets the responses of the host CSPs and sends their spot prices back to the VM manager
8. The VM manager examines the spot prices of the host CSPs and asks the adapter to register a new VM on the host CSP with the lowest spot price
9. The adapter sends the VM creation request to the specified host CSP. The host CSP creates the VM for the parasite CSP and sends the VM's identifier back to the adapter, which will send it to VM manager in turn
10. The VM manager stores the customer-VM assignment into the database. The VM's identifier is then sent back to the API, the GUI and the customer in order

## 3 Mathematical analysis

In this section, we first discuss about resource demands in CSPs and the need for extra resource provisioning. Then we deal with the parasite CSP and its (virtual) capacity. Finally, we will take the parasite CSP's SLA into account.

### 3.1 Mathematical background

Here we introduce the concept of *martingales*. They are used to deal with time-varying values with bounded variations in each time unit. We will use martingales to analyze the load of the data center.

The below definition and theorems are extracted from [3].

Definition 2 **Martingale**

A sequence $X_{0},\cdots X_{n}$ of random variables is a martingale if

$$E[X_{i}|X_{0},\cdots X_{i-1}]=X_{i-1}$$

for all 1≤i≤n.

The below theorem tells how to create a martingale from a sequence of random variables. Later, we will assume the load of the data center in each time as a random variable and leverage this theorem to create a martingale.

Theorem 1 *Let* $Z_{0},\cdots Z_{n}$ *be a sequence of random variables and*

$$X_{i}=E[Z_{n}|Z_{0},\cdots Z_{i}]$$

*for all* 0≤i≤n*. The sequence* $X_{0},\cdots X_{n}$ *is a martingale.*

The following theorem bounds the long-term variation of a martingale. Later in the paper, we use this theorem to bound load variations of the data center during the time.

Theorem 2 **Azuma's inequality**

*If the sequence* $X_{0},\cdots X_{n}$ *is a martingale and* s>0 *is a constant such that*

$$\left|X_{i}-X_{i-1}\right|\leq s$$

*for all* 1≤i≤n*, then*

$$Pr[\left|X_{i}-X_{0}\right|\geq \lambda s\sqrt{i}]\leq 2e^{-\lambda^{2}/2}$$

*for every* λ>0 *and* 0≤i≤n*.*

Chernoff bounds are used to bound the variation of sum of random variables from its mean. Assuming the unavailability of the CSP in each time being a random variable and its total unavailability in long-term being the sum of those random variables, we will use the following theorem later in the paper to bound the probability of SLA violation due to lack of availability.

Theorem 3 **Chernoff bound**

*A Poisson trial is a random variable* $X_{i}$ *whose value is* 1 *with probability* $p_{i}$*,* 0 *otherwise. If X is the sum of independent Poisson trials and* μ=E[X]*, then*

$$Pr[X>(1+\delta )\mu ]<{\left[\frac{e^{\delta }}{{\left(1+\delta \right)}^{1+\delta }}\right]}^{\mu }$$

*for all* δ>0*.*

### 3.2 Extra loads in CSPs

Assume that the CSP has a periodic schedule for buying or retiring servers and let *T* be the length of this period. At the beginning of each time interval, the CSP knows the current total demand of its customers, which is the sum of their individual demands. We assume that the demand can be represented by a non-negative integer.

At each time unit, the demand may be increased or decreased by some amount, due to new VMs requested by customers or resizing or terminating the current VMs. We assume that the variation of the demand in each time unit is bounded by a not so large number. This is reasonable, since a burst of large requests can be a notion of some availability attack and the CSP may not accept the whole burst. In addition, if multiple requests are hired by a customer adding to a large amount of virtual resources, the customer should have enough patience to wait for several time units for all his requested VMs to be provisioned. Of course, this variation limit is not constant and increases as the CSP becomes larger. We assume this limit to be *sμ* where *μ* is the expected total load of the CSP and *s* is a constant. In section 4.1 we will evaluate this assumption and investigate a reasonable value for the coefficient *s*.

Let $L_{t}$ (for 0≤t≤T) be a random variable denoting the load of the CSP at the end of the *t*-th time unit (for instance, $L_{T}$ is the load at the end of the time interval) and let $X_{t}$ be the expected value of $L_{T}$ after the *t*-th time unit:

$$X_{t}=E[L_{T}|L_{0},\cdots L_{t}]$$

We also define *μ* to be the expected value of $L_{T}$ at the beginning:

$$\mu =X_{0}=E[L_{T}|L_{0}]$$

Theorem 4 *For every* λ>0 *we have*

(1) $$Pr[\left|L_{T}-\mu \right|\geq \lambda s\mu \sqrt{T}]\leq 2e^{-\lambda^{2}/2}$$

Proof By Theorem 1, the sequence $X_{0},\cdots X_{T}$ is a martingale. From the fact that the deviation of loads in each time unit is at most *sμ*, we can apply Theorem 2 and get

$$Pr[\left|X_{t}-\mu \right|\geq \lambda s\mu \sqrt{t}]\leq 2e^{-\lambda^{2}/2}$$

for every λ≥0 and 0≤t≤T. By setting t=T and noting that $X_{T}=E[L_{T}|L_{0},\cdots L_{T}]=L_{T}$, inequality (1) results. □

Theorem 4 states that the load of the CSP at the end of the time interval is relatively close to its expectation with high probability. Define the *risk factor* of the CSP to be the probability of the CSP being out of resources at the end of the time interval. More formally, having a risk factor of ε>0, the CSP should procure additional resources that cover the demand at the end of the time interval with probability at least 1−ε. The smaller the risk factor of the CSP, the more the additional resources to be provisioned.

Theorem 5 *If the risk factor of a CSP is ε, then*

$$Pr\left[\left|L_{T}-\mu \right|\geq s\mu \sqrt{2T\ln\frac{2}{\varepsilon }}\right]\leq \varepsilon$$

Proof Results from (1) by setting the right-hand side to *ε*. □

We can define the amount of required additional resources as a function of *s* and *ε*:

(2) $$\mathrm{ext}(s,\varepsilon )=s\mu \sqrt{2T\ln\frac{2}{\varepsilon }}$$

So, Theorem 5 states that if the CSP's risk factor is *ε*, then it needs to provision at most ext(s,ε) additional resources.

On the other side, the load of the CSP may become less than *μ*. In fact, Theorem 4 states that if the CSP has provisioned ext(s,ε) additional resources, then the amount of free resources will be at most 2ext(s,ε) with probability at least 1−ε. This amount may become relatively large for small values of *ε* and this motivates the concept of spot prices.

### 3.3 Adding a parasite CSP to the ecosystem

Recall that ${\mathrm{pr}}_{o}$ and spot(l) are the on-demand and spot price for the host CSP respectively, where *l* is the current load of the CSP. The fact that the value of spot(l) is less than ${\mathrm{pr}}_{o}$ with a high probability is the main motivation for the concept of a parasite CSP that we introduced before.

Let *v* be the amount of resources that the parasite CSP has rent from the host CSP, *π* be its bid, $\varepsilon_{o}$ be the probability of the host CSP being unavailable and $\varepsilon_{p}$ be the probability of the spot price being above *π*. As discussed before, the parasite CSP becomes unavailable if either the host CSP becomes unavailable or the spot price exceeds the bid. So, if $\varepsilon_{u}$ is the unavailability probability of the parasite CSP, we can write

$$\varepsilon_{u}\leq \varepsilon_{o}+\varepsilon_{p}$$

as $\varepsilon_{o}$ is out of our control, we should control $\varepsilon_{u}$ by keeping $\varepsilon_{p}$ sufficiently small.

Choosing a relatively large value for *π* and a small value for $\varepsilon_{p}$, we want to know how large the value of *v* can be (i.e. how much resources can the parasite CSP rent). In fact, increasing the value of *v* increases the probability of the spot price being above *π*, so *π* should be increased in order to keep $\varepsilon_{p}$ small. However, choosing a very large *π* is a risk for the parasite CSP and is not desired.

In the following theorem, we obtain *v* as a function of *π* and $\varepsilon_{p}$. Our calculations depend on a proper estimation of *s*, which determines the maximum deviation of load in a time unit. However, as we will see soon, this value is just a scale factor in our equations.

Theorem 6 *If the parasite CSP rents an amount*

(3) $$v(\pi ,\varepsilon_{p})={\mathrm{spot}}^{-1}(\pi )-\mu -s\mu \sqrt{2T\ln\frac{2}{\varepsilon_{p}}}$$

*of resources from the host CSP, then the probability of the spot price being above π is no more than* $\varepsilon_{p}$*.*

Proof Fix a value for $\varepsilon_{p}$. In fact, if *X* is a random variable denoting the CSP's current load (excluding the load added by the parasite CSP), we should have

$$Pr[\mathrm{spot}(X+v)>\pi ]\leq \varepsilon_{p}$$

Since the spot price is a strictly increasing function, the above means

$$Pr[X>{\mathrm{spot}}^{-1}(\pi )-v]\leq \varepsilon_{p}$$

Using the result of (2) it suffices to have

$${\mathrm{spot}}^{-1}(\pi )-v=\mu +\mathrm{ext}(s,\varepsilon_{p})=\mu +s\mu \sqrt{2T\ln\frac{2}{\varepsilon_{p}}}$$

Rewriting the above with respect to *v* yields (3). □

Note that (3) expresses the virtual capacity of the parasite CSP over a single host CSP. Assume there exist *k* host CSPs with identical configurations but independent loads. In this case, when the spot price of a CSP exceeds *π*, the parasite CSP can migrate its VMs to another CSP. So, $\varepsilon_{p}$ becomes the probability that all host CSPs' spot prices exceed *π*. To this end, it suffices that the probability of each host CSP's spot price being above *π* be $\varepsilon_{p}^{1/k}$. The following corollary summarizes this result.

Corollary 1 *If the parasite CSP relies on k independent and identical host CSPs, then its virtual capacity can be*

(4) $$\text{virtual capacity}=v(\pi ,\varepsilon_{p}^{1/k})$$

Note that this setting is suboptimal and assumes that all of the parasite CSP's VMs rely on a single host CSP in each time. But this assumption is unnecessary and the possible capacity for the parasite CSP is much more.

Concluding this subsection, if the parasite CSP chooses a bid *π*, then it can set its (virtual) capacity to $v(\pi ,\varepsilon_{p})$ and be sure with probability $1-\varepsilon_{p}$ that the spot price will not exceed *π*. Note that the parasite CSP does not need to request all these resources at the beginning; it can request the resources or release them exactly when its customer requests or releases some (virtual) resources.

### 3.4 Service Level Agreement (SLA)

Cloud service providers usually expose an SLA as a guarantee for their quality of service. The SLA is verified in time intervals of length $T^{\prime }$ and consists one or more items whose general form is: “If the number of unavailable time units during the interval is at least *w*, the CSP must pay a penalty of *rq* to the customer, where *q* is the amount paid by the customer to the CSP”. Here, $T^{\prime }$ is the SLA verification period, *w* is the unavailability threshold and *r* is the penalty coefficient.

It's reasonable for the parasite CSP to define a number of such SLAs for its quality of service. Recall that the unavailability of the parasite CSP's service can be due to two different events (index *t* represents a time unit during the interval, $0\leq t<T^{\prime }$):

• the host CSP's service is unavailable at time unit *t* (event $E_{1t}$). Let $U_{1t}$ be a random variable indicating this event ($U_{1t}=1$ if the service is unavailable, 0 otherwise).
• the spot price of the host CSP is above *π* at time unit *t* (event $E_{2t}$). We can define random variable $U_{2t}$ in a similar way as above

Let *Z* be a random variable denoting the number of unavailable time units during the interval. We have

$$Z\leq \sum_{t=0}^{T^{\prime }-1}U_{1t}+U_{2t}$$

Recall that we denoted by $\varepsilon_{u}$ the unavailability probability of the parasite CSP. Here we observe that $\varepsilon_{u}=Pr[E_{1t}\cup E_{2t}]$. Choosing a sufficiently large value for *π* and assuming a highly available host CSP, the value of $\varepsilon_{u}$ is very small. But how about *Z*? Here, we propose a pessimistic as well as an optimistic upper bound for the penalty of SLA violation (i.e. having Z≥w).

Theorem 7 **Pessimistic penalty upper bound**

*The expected penalty of the parasite CSP is at most*

(5) $$\frac{r\varepsilon_{u}T^{\prime }}{w}$$

Proof Let $\mu_{Z}=\varepsilon_{u}T^{\prime }$ be the expectation of *Z*. Suppose we have monitored the parasite CSP's unavailability for *n* intervals and let $Z_{i}$ be the number of unavailable time units in the *i*-th interval.

We know

$$\mu_{Z}=\frac{\sum_{i=1}^{n}Z_{i}}{n}$$

Consider the worst case, in which exactly *k* of $Z_{i}$s are equal to *w* and all the others are zero. Hence

$$\mu_{Z}=\frac{kw}{n}$$

and we have

$$\frac{k}{n}=\frac{\mu_{Z}}{w}$$

The expected penalty in this case is rk/n (*k* penalties during *n* periods) and

$$\frac{rk}{n}=\frac{r\mu_{Z}}{w}=\frac{r\varepsilon_{u}T^{\prime }}{w}$$

Since this is the worst case, an upper bound is obtained for the expected penalty:

$$\text{expected penalty}\leq \frac{r\varepsilon_{u}T^{\prime }}{w}$$

as desired. □

Of course, the above analysis is too pessimistic. If we assume the unavailability of the parasite CSP to be independent in different time units, we can obtain a better upper bound.

Theorem 8 **Optimistic penalty upper bound**

*If events* $Pr[E_{1t}\cup E_{2t}]$ *are independent for different t, then the expected penalty will be at most*

(6) $$\text{expected penalty}\leq \frac{re^{w-\mu_{Z}}}{{\left(w/\mu_{Z}\right)}^{w}}={\left(\frac{\varepsilon_{u}T^{\prime }}{w}\right)}^{w}re^{w-\varepsilon_{u}T^{\prime }}$$

Proof Since events $Pr[E_{1t}\cup E_{2t}]$ are independent, they are independent Poisson trials and by Theorem 3 we have

$$Pr[Z>(1+\delta )\mu_{Z}]<{\left[\frac{e^{\delta }}{{\left(1+\delta \right)}^{1+\delta }}\right]}^{\mu_{Z}}$$

for all δ>0. By setting $\delta =(w/\mu_{Z})-1$ and multiplying by *r* the upper bound (6) results. □

The upper bound in (6) is much less than (5) for typical values of $\varepsilon_{u}$, $T^{\prime }$ and *w*, as we will see in subsection 4.5.

## 4 Evaluation

Our evaluation is performed on Amazon's EC2 services. This CSP rents its resources in an on-demand manner with a fixed price and rents its spare resources with a varying spot price.

In this section, we fix the values of our parameters as follows:

• We use minutes as time units, i.e. the variation of the host CSP's load in each minute is bounded by *sμ*, the spot price is calculated after each minute and the availability of the CSP is checked in each minute (for SLA)
• T=1440 is the number of minutes in a day, i.e. the host CSP has a daily schedule for buying or retiring its physical resources
• $T^{\prime }=43200$ is the number of minutes in a month, i.e. the SLA of the CSPs is verified monthly
• We deal with CPU cores as computational resources

### 4.1 Load variations in clouds

In section 3.2 we assumed that load variations of a cloud service provider can be bound by a not so large amount of *sμ*. Here, we evaluate this assumption using real data.

As we couldn't find any appropriate dataset for workloads in Amazon's EC2 services, we used another dataset for a distributed datacenter from BitBrains [4]. This dataset consists of different metrics of a number of VMs running on the datacenter. Metrics are measured in 5 minutes intervals in a one-month period and are presented in a set of CSV files, each row representing a single VM's metrics at a time. Here, we used the number of CPU cores of the VMs to observe the variations in the total workload of the datacenter. The number of CPU cores of a single VM may be zero at the beginning, indicating that the VM was not existed from the beginning and is created in the middle of the month. In contrast, most VMs have a positive number of CPU cores at the beginning which means that the VM was created at some time in the past. Similarly, some VMs have a positive number and some have a zero number of CPU cores at the end which indicates that the VMs are not yet removed or have been removed during the month, respectively. In addition, the number of CPU cores may be changed due to a resize.

Using the above dataset, we calculated the total load of the datacenter for each 5 minutes time interval, which is the sum of CPU cores for all VMs in that time interval. We observed that the maximum variation of load is 48 CPU cores in 5 minutes. It should be noted that the BitBrains data center is relatively small compared to Amazon's.

Also we analyzed a similar dataset for Microsoft Azure [5]. The data consists of creation and destroy time of a subset of Azure VMs as well as their sizes. The number of CPU cores of these VMs sums to about $3\times 10^{5}$ which can be identified as a sufficiently large data center. We observed that the maximum deviation of work load in this dataset is 3521 CPU cores in five minutes, which is also very small compared to data center size.

Although we couldn't find a formula for calculating *s*, setting its value to 0.002 seems to be reasonable regarding to the above two datasets. That is, we can assume that the change of load in each minute is at most 0.002*μ* in each minute, where *μ* is the average load of the data center. This value is used in calculations in later subsections.

### 4.2 More resources, less risk

In this subsection, we try to show the relation between the amount of additional resources procured by the CSP and the risk to be overprovisioned.

**Figure 2:**
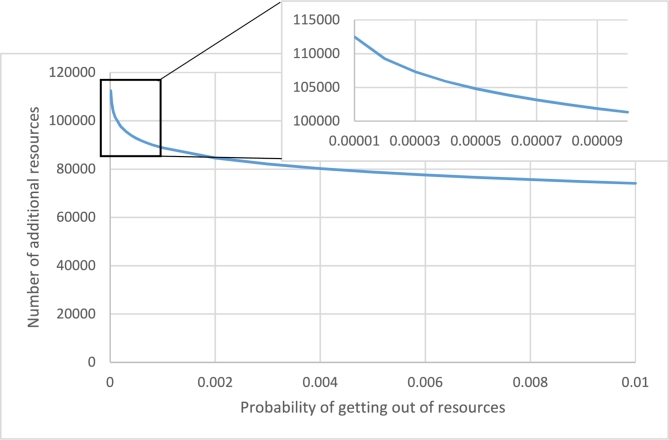
Number of additional resources for *s* = 0.002 and different *ε*.

Recall from subsection 3.2 that we defined the risk factor of the CSP to be the probability of being overloaded at the end of the day. Also recall from (2) that if the risk factor of the CSP is *ε* and the maximum deviation of its load in each minute is bounded by *sμ*, then it needs to procure an amount

$$\mathrm{ext}(s,\varepsilon )=s\mu \sqrt{2T\ln\frac{2}{\varepsilon }}$$

of additional resources. Fig. 2 shows this value for different values of *ε* and setting s=0.002, $\mu =3\times 10^{5}$ (Same as Azure dataset). Note that *s* is just a coefficient and the shape of the resulting curve is identical for different values of *s*.

For example, ext(0.002,0.0001)∼101331 is the number of additional resources for ε=0.0001. If the resources are CPU cores and each server has 32 CPU cores, this means that the CSP needs to buy 3167 additional servers in order to assure with probability 99.99% that it will not be overloaded at the end of the day. It can be seen that this value is relatively small and it's reasonable for a large CSP to procure this number of servers. Of course, many of these servers are in low-power mode most of the time and hence, the power consumption of the CSP is not proportionally increased.

### 4.3 Spot price by load

In this subsection, we try to investigate the spot price function of Amazon EC2 services. We retrieved the two-month trends (April and May, 2016) of spot price for its m3.2xlarge VMs each having 8 CPU cores and 32 GB of RAM [6]. We divided each price by 8 to obtain a price for each CPU core.

As we assumed the spot price to be a function of load, we need to estimate the current load of the data center. Let *L* be a random variable whose value is the current load. If we set s=0.002 (we will discuss about it at the end of this subsection) and ε=0.03 in (2), we have ext(0.002,0.03)∼95545 and hence

(7) Pr[|L−μ|>95545]≤0.03

From another viewpoint, the load of the data center is the sum of demands for the individual customers. If these demands have independent identical distributions, their sum can be estimated by a normal distribution $N(\mu ,\sigma_{n})$. So the current load of the data center can be estimated by a random variable $L_{n}$ having a normal distribution. By definition, the probability of having $\mu -3\sigma_{n}\leq L_{n}\leq \mu +3\sigma_{n}$ equals to $\mathrm{erf}(3/\sqrt{2})∼99.97\text{\%}$. So

(8) $$Pr[\left|L_{n}-\mu \right|>3\sigma_{n}]<0.03$$

Using (7) and (8) we can find a reasonable value for $\sigma_{n}$:

(9) $$\sigma_{n}=\frac{95545}{3}∼31848$$

Now let's define some useful functions ($2T^{\prime }=86400$ is the number of minutes in the two-month period, which is the length of our evaluation data):

• ts(t) is the spot price of Amazon EC2 at time *t* ($0\leq t<2T^{\prime }$)
• ps(p) is a value *x* such that the spot price is at most *x* with probability *p* (0≤p≤1). If we collect values of ts(t) for all $0\leq t<2T^{\prime }$ and sort them in an increasing order, then the $\left\lceil 2pT^{\prime }\right\rceil$-th element is the answer
• lp(l) is the probability of having L≤μ+l (for $-3\sigma_{n}\leq l\leq 3\sigma_{n}$). Using $L_{n}$ as an estimation of *L* we have
  $$lp(l)∼\frac{1}{2}\left[1+\mathrm{erf}\;\left(\frac{l}{\sigma_{n}\sqrt{2}}\right)\right]$$

Then we can estimate the spot price using the above functions:

spot(x)≈ps(lp(μ+x))

**Figure 3:**
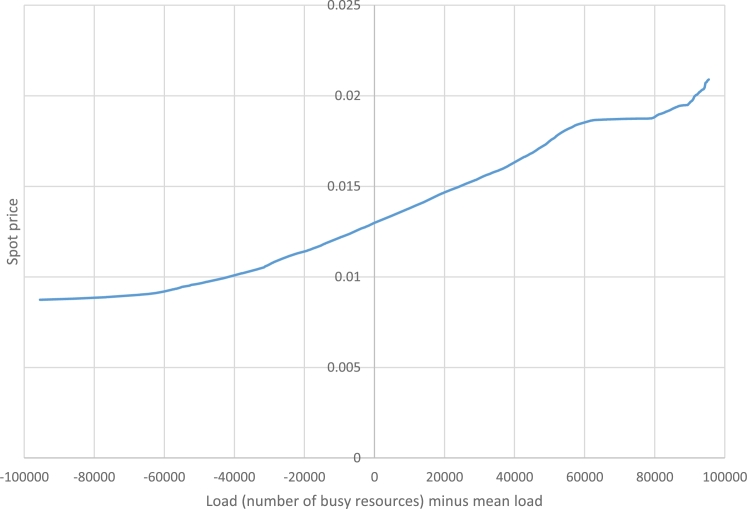
Estimation of spot prices as a function of data center load.

This function is represented in Fig. 3.

An important note in the above analysis is fixing s=0.002. What if the actual value of *s* is more or less than this value? Let's set s=0.002c where $c\in R^{+}$. We have

ext(0.002c,0.03)=c.ext(0.002,0.03)

$$\sigma_{n}^{\prime }=\frac{\mathrm{ext}(0.002c,0.03)}{3}=\sigma_{n}c$$

$$lp^{\prime }(l)=lp(lc)$$

$${\mathrm{spot}}^{\prime }(\mu +l)=\mathrm{spot}(\mu +lc)$$

where $\sigma_{n}^{\prime }$, $lp^{\prime }(.)$ and ${\mathrm{spot}}^{\prime }(.)$ correspond to $\sigma_{n}$, lp(.) and spot(.) respectively, when having s=0.002c.

We will use the spot function derived here in the subsequent subsections in order to analyze the gap between spot and on-demand prices.

### 4.4 Virtual capacity of the parasite CSP

In (4) we proposed a formula for estimating the virtual capacity of the parasite CSP. Here we try to calculate the virtual capacity using simulation.

As introduced in subsection 4.1, the BitBrains dataset contains information about work-load of a distributed data center. Since that data center is relatively small, we scale the work-load to generate the required simulation data. For this, we calculate the mean time between VM creation events, the mean time between VM destroy events as well as the distribution of VM sizes in those events. Using an exponential distribution to regenerate the events and then calculating the standard deviation of work-loads in each day, we scale the events until the standard deviation reach the value obtained in (9).

We generate this data for each of the *k* CSPs independently during one month period. Then the simulation begins to calculate the unavailability of the parasite CSP as a function of *v* – the amount of resources occupied. For each value of *v*, the simulation begins with renting *v* resources from the CSP having the minimum spot price (i.e. the most free CSP). During the time, if the amount of free resources of the host CSP becomes less than *v*, the parasite CSP migrates all its resources to another CSP, which has the least spot price at that time. In some times, all CSPs have less than *v* free resources and hence, none of them accept the parasite CSP. In these times, the parasite CSP cannot serve its users and becomes unavailable. Counting these unavailability events during the month and dividing by 43200 (the number of minutes in a month) gives the unavailability of the parasite CSP.

An important note here is migrating all VMs to the cheapest CSP. As noted in subsection 3.3, this assumption is made to ease mathematical analysis and in reality, a smooth migration plan is used instead of migrating all VMs at once. In addition, the parasite CSP should prevent downtimes during migrations. This results to a partitioning of VMs across CSPs as well as a replication mechanism to prevent downtimes. Dealing with this more complex system can be considered as a future work.

**Figure 4:**
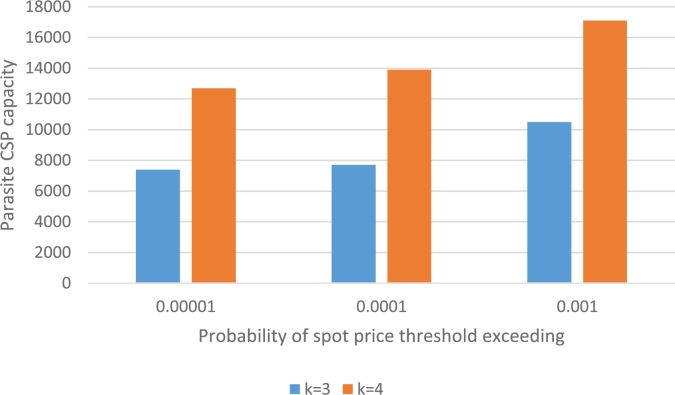
Virtual capacity of the parasite CSP by the bid-violation probability.

So we have a mechanism to calculate unavailability with respect to *v* and need the inverse; having an unavailability threshold $\varepsilon_{p}$ what is the maximum possible *v*? We use binary search to calculate this value which is represented in Fig. 4. It is worth noting that we used Python as programming language and run the simulation on a PC running Windows 10 with Intel Core i3 CPU.

As can be seen, the virtual capacity is considerable and makes the concept of a parasite CSP reasonable. However, the CSP should rely on several host CSPs so it can maintain its capacity at a high level while lowering the risk of unavailability due to large spot prices.

### 4.5 Service Level Agreement (SLA)

In this subsection, we investigate the ability of the parasite CSP to expose reasonable SLAs to its customers and the risk of their violation.

As discussed in 3.4, each SLA item can be specified by parameters *w* and *r*, which are the unavailability threshold and penalty coefficient, respectively.

Assume that the parasite CSP has exposed the same SLA as Amazon EC2, which can be summarized as follows:

• If the availability of the service in a month is below 99%, a penalty of 30% is paid back to the customer (w=433, r=0.3).
• If the availability is not below 99% but is below 99.95%, the penalty is 10% (w=22, r=0.1).

Define random variables $C_{1}$ and $C_{2}$ to be the penalties in a month for the two above items, respectively.

For example, suppose that we can keep the probability of unavailability in a minute to be at most 0.0011% (as in [7]). So $\varepsilon_{u}=0.000011$. For the first item, (5) yields:

$$E[C_{1}]\leq \frac{r\varepsilon_{u}T^{\prime }}{w}=0.03\text{\%}$$

and for the second item:

$$E[C_{2}]\leq \frac{r\varepsilon_{u}T^{\prime }}{w}=0.22\text{\%}$$

So the expected total penalty for this SLA is at most 0.25%, which is a bit large, but not so bad, regarding to our too pessimistic analysis.

If we use (6) for bounding the penalty, we can set ($\mu_{Z}=4.32$ is the expected number of unavailable minutes during a month):

$$\delta_{1}=\frac{0.01\times 43200}{4.32}-1=909$$

$$\delta_{2}=\frac{0.0005\times 43200}{4.32}-1=45$$

and the expected penalties are no more than

$$E[C_{1}]\leq 0.3\times Pr[Z>(1+\delta_{1})\mu_{Z}]<6.43\times 10^{-1095}$$

$$E[C_{2}]\leq 0.1\times Pr[Z>(1+\delta_{1})\mu_{Z}]<5.08\times 10^{-29}$$

summing to an upper bound of $5.1\times 10^{-29}$.

Table 1 shows optimistic as well as pessimistic upper bounds for the SLA violation penalty with respect to the availability commitment stated in the SLA. It is assumed that the expected availability is 99.9989%, from the results of [7].

**Table 1:** Optimistic and pessimistic probability of SLA violation for different availability commitments.

Commitment	Optimistic	Pessimistic
99%	2.14 × 10^−1094^	1.10 × 10^−3^
99.5%	8.05 × 10^−484^	2.19 × 10^−3^
99.9%	2.36 × 10^−68^	1.08 × 10^−2^
99.95%	5.08 × 10^−28^	2.16 × 10^−2^

The calculations provided and the numbers presented in the table show that SLAs are meaningful in parasite CSPs paradigm and even exposing the same SLAs as Amazon's makes sense.

### 4.6 On-demand price of parasite CSP

In this subsection, we investigate the question that whether the parasite CSP can expose an on-demand price less than that of the host CSP or not?

To answer the question, we make a comparison between the host CSP's on-demand price and the expected value of its spot prices when the parasite CSP exists. In fact, as we assume the spot price being a function of load, presence of the parasite CSP affects the price.

**Figure 5:**
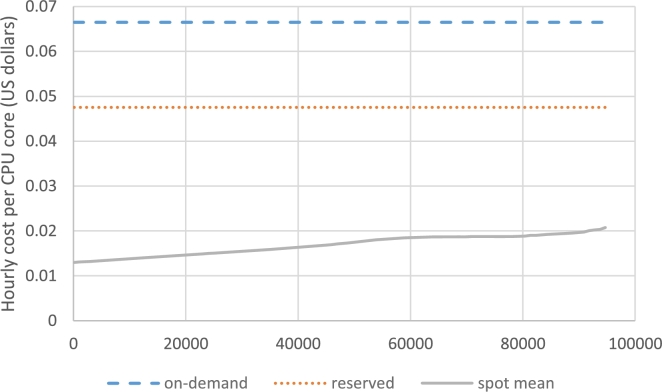
Mean spot price vs on-demand and reserved prices.

Fig. 5 shows the comparison. Recently, Amazon has removed m3.2xlarge VMs from the list and, as discussed in subsection 4.3, our data belongs to 2016. Hence, we consider that time's price of m3.2xlarge VMs for comparison; the on-demand price of the VM was 0.532$ hourly (that is, 0.067$ per CPU core) and the reserved price was 277.4$ monthly (that is, 0.048$ per CPU core hourly). These two numbers are represented in the figure by dashed and dotted horizontal lines, respectively.

For spot prices, we use the result of subsection 4.3. The mean value of the spot price equals to spot(μ+v) where *v* is the number of resources occupied by the parasite CSP and *μ* is the mean load of the host CSP without the parasite CSP. This value is represented by a solid curve in the figure.

From the figure, we observe that the mean spot price is much less than even the reserved price. In fact, the parasite CSP can choose any number between 0.03$ and 0.04$ as on-demand hourly price per CPU core to ensure that the value is far enough to both the host CSP's reserved price and the cost paid for the spot price.

Determining an exact value for the on-demand price of the parasite CSP is beyond this paper and can be done as a future work, but the important thing here is the possibility of determining such a value.

## 5 Related works

Considerable work has been done on the spot instances of the CSPs, specifically Amazon EC2 services. In this section, we provide an overview on the existing work in three categories.

The first category are research works that try to analyze and model price variations in Amazon EC2 spot instance prices. In [8], [9] traces of the spot prices are gathered and analyzed. They calculate statistical measures over the traces in different hours of day as well as different days of week. An estimation of the spot price function using a mixed Gaussian distribution is also proposed. Ben-Yehuda and Ben-Yehuda [10] analyze long-time traces of Amazon EC2 spot prices in different zones. They argue that although widely believed, the determination of spot prices is not totally market-driven and low spot prices are set by random. However, the higher spot prices are market-driven and are determined by user bids. Karunakaran and Sundarraj [11] use a simulation study on data gathered from Amazon EC2 to analyze the effect of increasing or decreasing the bid price on job completion cost, wait time and interruption rates during job execution. Li et al. [12] develop a Predator-Prey model for simulating market activities in order to explain variations in spot prices. They modeled demand and resource as predator and prey, respectively. They identify some regular patterns of market activities with respect to Amazon EC2 spot prices. Agarwal et al. [13] propose a method for forecasting Amazon EC2 spot prices based on recurrent neural networks. They argue that the error of their method is at most 8.6%. In [14], Baughman et al. presented a long/short-term memory (LSTM) recurrent neural network for spot price prediction, arguing the error being less than that of the ARIMA method. Portella et al. [15] propose static analysis over on-demand and spot prices of Amazon EC2 services. They capture the correlation between VM types and their on-demand price as well as spot price trends. With this information, they provide a price-availability tradeoff to the user. For instance, the user can set the bid to 30% of the on-demand price and ensure that the availability of the VM will be above 90%. Baughman et al. [16] recognize a major change in Amazon's spot price mechanism in 2017. They analyze spot prices before that time as well as current prices and compare some of their properties.

The above works try to model the spot prices and their variations. In fact, these works are orthogonal to ours and the concept of a parasite CSP can be imagined in all models. However, the details of the analysis depend on the model accepted for spot prices.

The second category of the existing work is about to optimize the behavior of the CSP about its spot instances. Zhang et al. [17] assume an auction-based model for exposing the VMs. They propose a mechanism that predicts the future demand for different VM types and then determines the optimum spot price as well as capacity for each VM type in order to maximize the CSP's profit. Toosi et al. [18] propose an auction-based mechanism for determining the price of perishable cloud resources. Their mechanism is envy-free, near optimal (in terms of profit) and is truthful with high probability.

The mentioned works can be interpreted as suggestions to the CSPs for their spot prices. As the previous category of related research, the behavior of the CSPs about the spot prices affect the details of our analysis, but the concept of a parasite CSP is meaningful in all cases.

Finally, some researchers try to leverage the spot instances of the existing cloud service providers and propose models and mechanisms for their external users to gain profit. Here, we introduce these works and compare them to ours.

Mattess et al. [19] discuss the idea of using spot instances in peak loads. Computing clusters having variable loads can rent the spot instances of an IaaS provider when a peak occurs in their load. They analyze different service provisioning policies in this context. We can interpret our work as a basis for theirs. In fact, the analysis presented in our paper can be used to better understand the possibility of using spot prices in peak loads.

Yi et al. [20], [21] propose and compare several checkpointing schemas when using spot instances of Amazon EC2 services, such as hourly, rising edge-driven and current-price based adaptive checkpointing. They also study the impact of work migrations on improving task completion times while maintaining low costs, by proposing and evaluating several migration heuristics. Compared to our work, although both try to rely on several CSPs to increase quality, the points of focus differ: They focus on how to migrate the work and we focus on how much resources can be rent. In fact, a future work can combine these works to obtain a more detailed picture of how a parasite CSP works.

[22] proposes a method for hosting an always-available service over the spot instances in order to reduce the relevant costs. It mainly consists of a scheduler that bids appropriately for spot prices in order to remain available and a mechanism for migrating the VMs from spot instances to on-demand instances when needed. Compared to us, they have focused on the migration and bidding mechanisms for increasing availability, while our focus is on the mathematical analysis of such availability in terms of SLA penalties and the number of resources we can rent.

[23] develops an information service named SpotLight that monitors the availability of different server types in different regions. Cloud applications can query this service to know about their server availability. Spot prices have an important role in their analysis. Their work can be used as a tool for deploying a parasite CSP. In fact, we assumed all information about spot prices and CSP availabilities can be accessed by the parasite CSP and SpotLight can be the tool to achieve this.

[7] develops a cloud platform named SpotCheck which provides IaaS on top of the spot instances of a native IaaS provider. The price of the provided service is near the spot price of the underlying CSP but its availability is about 99.9989%. This work is also related to ours in the manner that they try to create a tool that rents spare resources from a native provider and exposes them to its users. In comparison to that work, our contribution is to expose spare instances with a fixed price and also to calculate the virtual capacity of the parasite CSP. We used their availability result in our evaluations in subsection 4.5.

## 6 Conclusion

In this paper, we introduced the concept of parasite cloud service providers which can provide computational resources with an on-demand price less than the on-demand price of the typical CSPs. The main idea was to leverage the spare resources of one or several CSPs. We proposed the overall architecture of the parasite CSP as well as its model of interaction to the stakeholders.

We also analyzed the possibility of such CSPs existence; our analysis shows that a relatively large amount of spare resources is likely to exist in a typical CSP. We also showed that if the parasite CSP relies on several (even two) independent CSPs, it can assure its availability with high probability. The SLA of such CSP has been also analyzed and we obtained a pessimistic as well as an optimistic upper bound on its expected penalty of SLA violation. The optimistic upper bound is somewhat negligible and the pessimistic one is relatively small. So, our analysis shows that a parasite CSP can exist providing services with acceptable availability and exposing the same SLA as typical CSPs.

Several extensions to this work can be made as future works. The legal aspects of creating parasite CSPs can be studied. These aspects may vary among different countries or among different host CSPs. In fact, the basic model proposed in this paper can be refined or customized for different countries or host CSPs regarding to these legal aspects. Also recall that our model assumed all resources of the parasite CSP to rely on a single host CSP at each time so that it migrates the whole to another host CSP when needed. Hence, another extension is to consider partitioning the resources among several host CSPs or even replicate some VMs in order to be more available. Another extension is to propose mechanisms to prevent downtimes during migrations.

## Declarations

### Author contribution statement

Hamid Haghshenas: Conceived and designed the experiments; Performed the experiments; Analyzed and interpreted the data; Contributed reagents, materials, analysis tools or data; Wrote the paper.

Jafar Habibi: Conceived and designed the experiments; Contributed reagents, materials, analysis tools or data; Wrote the paper.

Mohammad Amin Fazli: Conceived and designed the experiments; Wrote the paper.

### Funding statement

This research did not receive any specific grant from funding agencies in the public, commercial, or not-for-profit sectors.

### Competing interest statement

The authors declare no conflict of interest.

### Additional information

No additional information is available for this paper.

## References

[1] Iankoulova I., Daneva M. (2012). Cloud computing security requirements: a systematic review. Proceedings of the Sixth International Conference on Research Challenges in Information Science (RCIS).

[2] Zhang Q., Cheng L., Boutaba R. (2010). Cloud computing: state-of-the-art and research challenges. J. Internet Serv. Appl..

[3] Motwani R., Raghavan P. (2010). Randomized Algorithms.

[4] Shen S., van Beek V., Iosup A. (2015). Statistical characterization of business-critical workloads hosted in cloud datacenters. Proceedings of the 15th IEEE/ACM International Symposium on Cluster, Cloud and Grid Computing (CCGrid).

[5] Cortez E., Bonde A., Muzio A., Russinovich M., Fontoura M., Bianchini R. (2017). Resource central: understanding and predicting workloads for improved resource management in large cloud platforms. Proceedings of the 26th Symposium on Operating Systems Principles.

[6] Monge D.A. (2018). Amazon web services (AWS) spot prices data 2016.

[7] Sharma P., Lee S., Guo T., Irwin D., Shenoy P. (2015). Spotcheck: designing a derivative IaaS cloud on the spot market. Proceedings of the Tenth European Conference on Computer Systems.

[8] Javadi B., Thulasiramy R.K., Buyya R. (2011). Statistical modeling of spot instance prices in public cloud environments. Proceedings of the Fourth IEEE International Conference on Utility and Cloud Computing (UCC).

[9] Javadi B., Thulasiram R.K., Buyya R. (2013). Characterizing spot price dynamics in public cloud environments. Future Gener. Comput. Syst..

[10] Agmon Ben-Yehuda O., Ben-Yehuda M., Schuster A., Tsafrir D. (2013). Deconstructing Amazon EC2 spot instance pricing. ACM Trans. Econ. Comput..

[11] Karunakaran S., Sundarraj R.P. (2015). Bidding strategies for spot instances in cloud computing markets. IEEE Internet Comput..

[12] Li Z., Tärneberg W., Kihl M., Robertsson A. (2016). Using a predator-prey model to explain variations of cloud spot price. Proceedings of the 6th International Conference on Cloud Computing and Services Science (CLOSER).

[13] Agarwal S., Mishra A.K., Yadav D.K. (2017). Forecasting price of Amazon spot instances using neural networks. Int. J. Appl. Eng. Res..

[14] Baughman M., Haas C., Wolski R., Foster I., Chard K. (2018). Predicting Amazon spot prices with LSTM networks. Proceedings of the 9th Workshop on Scientific Cloud Computing.

[15] Portella G., Rodrigues G.N., Nakano E., Melo A.C. (2019). Statistical analysis of Amazon EC2 cloud pricing models. Concurr. Comput., Pract. Exp..

[16] Baughman M., Caton S., Haas C., Chard R., Wolski R., Foster I., Chard K. (2019). Deconstructing the 2017 changes to AWS spot market pricing. Proceedings of the 10th Workshop on Scientific Cloud Computing.

[17] Zhang Q., Zhu Q., Boutaba R. (2011). Dynamic resource allocation for spot markets in cloud computing environments. Proceedings of the Fourth International Conference on Utility and Cloud Computing (UCC).

[18] Toosi A.N., Vanmechelen K., Khodadadi F., Buyya R. (2016). ACM Trans. Auton. Adapt. Syst..

[19] Mattess M., Vecchiola C., Buyya R. (2010). Managing peak loads by leasing cloud infrastructure services from a spot market. 12th International Conference on High Performance Computing and Communications (HPCC).

[20] Yi S., Kondo D., Andrzejak A. (2010). Reducing costs of spot instances via checkpointing in the Amazon elastic compute cloud. Proceedings of the 3rd International Conference on Cloud Computing.

[21] Yi S., Andrzejak A., Kondo D. (2012). Monetary cost-aware checkpointing and migration on Amazon cloud spot instances. IEEE Trans. Serv. Comput..

[22] He X., Shenoy P., Sitaraman R., Irwin D. (2015). Cutting the cost of hosting online services using cloud spot markets. Proceedings of the 24th International Symposium on High-Performance Parallel and Distributed Computing.

[23] Ouyang X., Irwin D., Shenoy P. (2016). Spotlight: an information service for the cloud. Proceedings of the 36th International Conference on Distributed Computing Systems (ICDCS).

